# Editorial: Aqueous-Phase Catalytic Conversions of Renewable Feedstocks for Sustainable Biorefineries

**DOI:** 10.3389/fchem.2020.629578

**Published:** 2020-12-15

**Authors:** Georgios Papadogianakis, Roger A. Sheldon, Dmitry Yu. Murzin, Yulong Wu

**Affiliations:** ^1^Industrial Chemistry Laboratory, Department of Chemistry, National and Kapodistrian University of Athens, Athens, Greece; ^2^Molecular Sciences Institute, School of Chemistry, University of the Witwatersrand, Johannesburg, South Africa; ^3^Department of Biotechnology, Delft University of Technology, Delft, Netherlands; ^4^Laboratory of Industrial Chemistry and Reaction Engineering, Abo Akademi University, Biskopsgatan, Finland; ^5^Laboratory of Advanced Reactor Engineering and Safety of Ministry of Education, Institute of Nuclear and New Energy Technology, Tsinghua University, Beijing, China

**Keywords:** water, aqueous medium, green and sustainable catalysis, biomass, lignocellulose, biorefinery, biofuels, platform chemicals

Today, a major research field with enormous growth potential is green catalytic conversions of renewable biomass and its downstream products which decisively contributes to the transition from a fossil-based society into a carbon neutral, sustainable, bio-based economy. Especially interesting is the topic of aqueous-phase catalytic conversions of renewable biomass-based feedstocks in biorefineries to manufacture liquid biofuels, commodity chemicals, value added chemicals and new biomaterials. (i) As a polar solvent water is the ideal medium to convert polar biomass and its upgraded products, (ii) it is involved either as a reagent or as a byproduct in biomass valorization, (iii) it could act as a catalyst in liquefaction subcritical reactions, (iv) it has a beneficial effect and boosts rates in several types of reactions such as hydrogenations and aldol condensations, (v) it possesses a large heat capacity which makets it a suitable medium to perform safely large scale exothermic reactions, (vi) it is a non-toxic, non-inflammable, ubiquitous, inexpensive, abundantly available, green, and sustainable solvent.

The conversion of feedstocks in a biorefinery will largely involve different processes to those involved in a petrochemical refinery. In the latter feedstocks are gaseous or liquid hydrocarbons that are oxidized at elevated temperatures, in the vapor or liquid phase, under solvent-free conditions. In contrast, the feedstocks in biorefineries consist of solid, water soluble carbohydrates and their conversion involves, *inter alia*, aerobic oxidations of hydroxyl functional groups, in water as solvent, under relatively mild conditions. This requires the development of environmentally attractive and cost-effective processes, employing both chemo- and biocatalytic technologies as discussed in the review by Sheldon. Biomass platform chemicals build a bridge from the biomass to commodity chemicals as a part of sustainable utilization. These biomass platform chemicals derive from the primary biomass such as lignin and cellulose through different routes and are converted into more refined chemicals through a variety of pathways. The review article of Wu et al. summarizes the recent research progress on dehydration, hydrogenation, oxidation and other reactions of platform chemicals by heterogeneous catalysts in the aqueous phase. Levulinic acid is classified as a key platform chemical for biorefineries, due to its large spectrum of potential applications and because it is readily available from lignocellulose by low-cost and high-yield production routes. The review article by Papadogianakis et al. focuses on recent advances in ruthenium-catalyzed hydrogenations of levulinic acid in aqueous medium using heterogeneous catalysts on solid supports, water-dispersible catalytic nanoparticles and homogeneous water-soluble catalytic complexes with biphasic catalyst separation, for the manufacture of advanced biofuels, value-added chemicals, sustainable solvents, additives to gasoline and to food. The significance of the aqueous solvent to carry out catalytic hydrogenations of levulinic acid has been highlighted in numerous experimental investigations and several theoretical studies. The utilization process of renewable resources involves a series of transformation processes, and many important intermediate products are produced in these processes. Adipic acid is an example of such an important bulk chemical. Due to the generation of toxic by-products in the existing processes, it has become an urgent problem to find environmentally friendly alternative pathways to the synthesis of adipic acid. The oxidation of glucose, has the potential to be an alternative route to the current oxidation of cyclohexanone/cyclohexanol mixtures in a petrochemical refinery. Jin et al. report in the mini review article on the progress made in the oxidation reactions of the above mentioned route to adipic acid.

Among several key aspects of biomass conversion in the aqueous phase for production of biofuels and chemicals several authors of this special collection of articles considered primary transformations of cellulose and hemicellulose as well as downstream treatment. Depolymerization of cellulose under milder conditions involving hydrolytic hydrogenation with formation of hexitols and hydrogenolysis leading to glycols was reported in the mini review by the late E. Sulman et al. The authors discussed mainly the promising catalytic systems and operation conditions clearly indicating the challenges on the path to industrial implementation of this technology as a part lignocellulose biorefinery to value-added products. The review of Xin et al. besides the synthesis of polyols through hydrolytic hydrogenation and diols by selective hydrogenolysis covers also hydrogenolysis and hydrodeoxygenation to alkanes as well as dehydration of fructose and subsequent oxidation reactions using cellulose as a starting material in aqueous medium. The authors discussed mechanistic aspects of these transformations and listed the remaining challenges and research objectives. In recent years, a variety of solvents mixed with water have been developed for lignocellulose valorization reactions. The H_2_O/THF system is one of such commonly mixed solvents. It has shown excellent properties in increasing the solubility and fractionation of lignin, cellulose and hemicellulose. Due to the extraction effect of THF on organic products, it can promote the reactions and the directional selection of products. The review by Hu et al. discusses and summarizes the above characteristics of the H_2_O/THF system.

An envisaged key platform chemical for the production of chemicals and liquid fuels in a biorefinery is 5-hydroxymethylfurfural (5-HMF) which can be produced by acid-catalyzed dehydration of fructose or, preferably, glucose. Unfortunately, this process is plagued by relatively low yields and high production costs that are largely a result of the low stability of 5-HMF under the acidic reaction conditions. In the original research article by Tongtummachat et al. substantial improvements were obtained by conducting the reaction in continuous operation in a biphasic methyl isobutyl ketone/water system in a dispersed flow reactor. A 5-HMF yield of 81.7% and a selectivity of 89.8% were obtained at a reaction temperature of 180°C and a residence time of 3 min. In another approach, Held et al. investigated the dehydration of fructose at 50°C in deep eutectic solvents (DES) consisting of mixtures of tetraethylammonium chloride or choline chloride with lactic or levulinic acid and a vanadium-containing heterpoly acid (H_8_PV_5_Mo_7_O_40_) as the catalyst. Using the latter, in combination with tetraethylammonium chloride and levulinic acid, afforded a 5-HMF yield of 57% and a selectivity of ca. 70% after 5 h reaction time.

Srinivasan et al. examined the hydrogenation of sugars such as xylose, glucose, and mannose to obtain quantitative selectivities toward their corresponding sugar alcohols xylitol, sorbitol, and mannitol using hydrous ruthenium oxide catalyst precursors supported on Na-β zeolite in aqueous solvent. Recycling experiments have shown that this catalyst is stable without losing its activity for five successive runs. Luque et al. developed nanocatalysts functionalized by yttrium oxide for the efficient mono-dehydration reaction of sugar alcohols such as sorbitol and mannitol under mild reaction conditions i.e., at room temperature to obtain with high selectivity the mono-dehydration products in aqueous media. Mechanistic studies indicated that the high efficiency for the mono-dehydration reaction is based mainly on the stability of the catalytically active intermediate species during the dehydration reaction.

An alternative to heterogeneous catalytic depolymerisation of lignocellulosic materials to sugars is enzymatic hydrolysis which is known to be impaired by generated sugar inhibition. The original research work of Ottens et al. presents strategies on conducting extractive enzymatic hydrolysis in aqueous two-phase systems paving the way to eventual design of a feasible process. High content of oxygen in lignocellulosic biomass requires not only efficient processes for deoxygenation quite often using hydrogen, but also sustainable routes for production of biohydrogen from renewable sources. One particular aspect of this was considered by Lefferts et al. who in an original research article reported aqueous phase reforming of butanol over rhodium supported on zirconia, specifically addressing the important issue of the influence of internal mass transfer on activity and selectivity. In the original research work of Özşen depolymerisation of lignocellulosic biomass, namely hazelnut shell waste, was discussed through application of hydrothermal liquefaction under supercritical conditions. The main focus of the author was on production of levulinic acid. Another very interesting aspect of the work was related to a combination of hydrolysis and electrolysis in subcritical water.

## Author Contributions

All authors listed have made a substantial, direct and intellectual contribution to the work, and approved it for publication.

## Conflict of Interest

The authors declare that the research was conducted in the absence of any commercial or financial relationships that could be construed as a potential conflict of interest.

